# Liver Failure of Wilson's Disease With Manifestations Similar to Porphyria and Uncommon *ATP7B* Gene Mutation: A Case Report and Literature Review

**DOI:** 10.3389/fmed.2021.702312

**Published:** 2021-07-26

**Authors:** Ju Zou, Ying-Hao Wang, Ling Wang, Ruo-Chan Chen

**Affiliations:** ^1^Department of Infectious Disease, Xiangya Hospital, Central South University, Changsha, China; ^2^Hunan Key Laboratory of Viral Hepatitis, Xiangya Hospital, Central South University, Changsha, China; ^3^Department of Ophthalmology, The Second Xiangya Hospital, Central South University, Changsha, China

**Keywords:** ATP7B, copper storage disease, liver failure, porphyria, Wilson's disease

## Abstract

**Background:** Wilson's disease (WD) is a rare condition; its diagnosis is challenging owing to a wide spectrum of *ATP7B* genotypes and variable clinical phenotypes, along with environmental factors. Few cases of WD with presentation of skin lesions and acute neurovisceral symptoms have been reported in the literature. To our knowledge, this is the first reported case of WD with an uncommon *ATP7B* gene mutation and rare symptoms of photosensitivity, sensation abnormality, and skin eruption occurring in a 19-year-old woman.

**Case presentation:** We report the case of a 19-year-old woman with WD presenting with liver failure, skin manifestations, and acute neurovisceral symptoms.The rare mutation in intron 1 of ATP7B (c.51+2T > G) was further confirmed by gene sequencing. The patients' symptoms improved after administration of penicillamine and zinc therapy combined with plasma exchange. She received long-term penicillamine treatment, and her liver function was within the normal range at 1 year after discharge. However, she underwent liver transplantation at 1.5 years after discharge.

**Conclusions:** We present a case of WD with a novel *ATP7B* gene mutation that may serve as a reference to generalists and specialists in hepatology or neurology of the rare clinical characteristics of WD, to prevent misdiagnosis and aid in the early diagnosis and treatment of the condition.

## Introduction

Wilson's disease (WD), also termed hepatolenticular degeneration, was first reported by Kinnear Wilson in 1912. WD is an autosomal recessive inherited disease, which is caused by a dysfunction of the *ATP7B* gene located on chromosome 13q14.3 (~80 kb). The *ATP7B* gene is composed of 21 exons and 20 introns encoding a copper-transporting P-type ATPase containing 1,465 amino acids that comprise six copper binding domains, eight transmembrane domains, and one ATP loop (1). It plays a key role in transforming apoceruloplasmin into ceruloplasmin and evacuating excessive copper into biliary canaliculi. Defects of ATP7B will result in an overdose of copper accumulation in many vital organs, primarily in the liver and brain (2). The incidence of WD is between 1 in 30,000 and 1 in 100,000 people worldwide (3). However, reports of the disease occurring in the Chinese population have been increasing (4).

Additionally, patients with WD present with variable clinical manifestations, including hepatic abnormality and neurological and psychiatric disturbances. Liver damage includes decompensated chronic liver disease, chronic hepatitis, and fulminant hepatic failure, leading to severe coagulopathy, infections, hepatorenal syndrome, and hepatic encephalopathy. However, less common clinical features include renal disease, osteoarthritis (5), and pancreatitis (6). Some reports have shown that different *ATP7B* mutation genotypes cause distinctive clinical phenotypes (7). Consequently, WD is easily misdiagnosed as other diseases owing to the heterogeneity of its clinical presentations and loci mutations.

Porphyria, a term for a group of metabolic diseases, leads to an overload of porphyrins and their precursors. Acute intermittent porphyria (AIP) is characterized by acute neurovisceral symptoms and abdominal pain predominantly (8), while porphyria cutanea tarda (PCT) is characterized by the development of blisters and sores after light exposure (9). WD presenting originally as skin lesions and acute neurovisceral symptoms similar to AIP or PCT has been rarely reported.

Here, we report a case of a young Chinese woman with WD and an uncommon *ATP7B* gene defect, similar to porphyria.The patient presented with photosensitivity of rash, paresthesia, acute neurovisceral symptoms of stomachache, and exacerbation of liver failure. Additionally, we present relevant literature to discuss our conclusions.

## Case Presentation

A 19-year-old woman was admitted to the Department of Infectious Diseases, Xiangya Hospital of Central South University on September 14, 2019, with the chief complaint of body numbness for 9 days and stomachache with jaundice for 5 days. She developed acute burning sensations on the skin of the forehead, face, neck, and palms of both hands that progressed into numbness, especially in the proximal joints on September 5, 2019. The above symptoms progressively worsened, with blisters and pruritus on sun-exposed areas. She experienced paroxysmal colic in the upper right abdomen, with no nausea, vomiting, fever and diarrhea on September 11, 2019, and presented with jaundice gradually. After a series of treatments, including anti-inflammatory, antispasmodic, liver protective, and rehydration medications at the local hospital, the symptoms of abdominal pain were relieved, jaundice was intensified and the total bilirubin level continued to rise (36.67 mg/dL). She was then referred to our hospital for further treatment.

During her pregnancy in February, 2019, the patient was diagnosed with abnormal liver function and took ursodeoxycholic acid (0.25 mg TID) irregularly, resulting in the continued increase of her aspartate aminotransferase (AST) and alanine aminotransferase (ALT) levels. In July, 2019, cesarean delivery was performed on the primigravida at 36 weeks and 5 days of gestation because of misdiagnosis of intrahepatic cholestasis of pregnancy at a local hospital. The baby boy's weight was 2.7 kg, with an Apgar score of 10. The patient had no history of drug abuse or known allergies, and had never smoked or drank alcohol. She had no history of liver disease or similar symptoms in her families. Notable examination findings included anemia, severe jaundice, liver palms, hepatosplenomegaly, belly bulge, and shifting dullness. Remarkably, some scattered skin rashes with pigmentation were present on her scalp and forehead.

After the patient was admitted, laboratory findings revealed mild erythropenia, moderate hypochromia, and significantly elevated total bilirubin (27.13 mg/dL) and aminotransferase (AST > ALT) levels. Poor hepatic synthetic function revealed low levels of total protein (58.5 g/L) and albumin (32.9 g/L). Additionally, prothrombin time was prolonged and prothrombin activity was severely decreased to 34.44% (Table 1). Severe and prominent liver damage was observed in this patient, whereas hepatitis B virus, autoimmune hepatitis-related antibodies, and Budd-Chiari syndrome were ruled out, and the patient had no history of alcohol and use of drugs as treatment for liver lesions. Inherited metabolic diseases were considered first because of the young age of onset with obvious liver enlargement and cirrhosis on abdominal ultrasound examination. Porphyria was suspected based on the initial symptoms of photosensitivity, acute stomachache, and pink urine under Wood's lamp illumination. However, genetic sequencing without porphyria-associated gene mutations excluded the diagnosis.

**Table 1:** Laboratory results of the patient at the first day of admission and 1.5 years after discharge and a data comparison of another WD patient with a different mutation.

**Laboratory tests**	**The patient of this case**		**The control patient**
	**First day of admission**	**1.5 years after discharge (recently)**	**First day of admission**
WBC ( × 10^9^/L) (3.5–9.5)	6.6	9.24	1.9
N ( × 10^9^/L) (1.8–6.3)	5.0	4.86	1.4
RBC ( × 10^12^/L) (3.80–5.10)	2.55	5.15	4.36
Hb (g/L) (115–150)	89	145	127
PLT ( × 10^9^/L) (125–350)	174	288	17
ALT (U/L) (7–40)	42.5	24.7	17.6
AST (U/L) (13–35)	61.7	19.6	26
Γ-GGT (U/L) (7–45)	157.6	52.3	104.7
TB (μmol/L) (1.7–17.1)	464.0	9.6	8
CB (μmol/L) (0–6.8)	255.8	2.9	4.7
TP (g/L) (65–85)	58.5	71	63.1
Alb (g/L) (40–55)	32.9	45.2	40.1
PT (s) (10–16)	23.7	12.4	14.4
PTA (%) (70–140)	34.44	119	82
INR (0.8–1.2)	1.91	0.91	1.15
APTT (s) (20–43)	70.9	36.5	39.7
Fibrinogen (g/L) (2–4)	1.47	2.88	2.1
C3 (mg/L) (790–1,520)	254	n.a.	612
C4 (mg/L) (100–400)	79.8	n.a.	142
Ceruloplasmin (mg/L) (210–530)	66.1	n.a.	33.2
24-H urine copper (μg/24 h) (15–30)	3,804	n.a.	2,899
Coombs test (–)	–	n.a.	n.a.

*n.a., not available; WBC, white blood cells; N, neutrophils; RBC, red blood cells; Hb, hemoglobin; PLT, platelets; ALT, alanine aminotransferase; AST, aspartate transaminase; γ-GGT, gamma-glutamyltransferase; TB, total bilirubin; CB, conjugated bilirubin; TP, total protein; Alb, albumin; PT, prothrombin time; PTA, prothrombin activity; INR, international standard ratio; APTT, activated partial thromboplastin time*.

**Figure 1:**
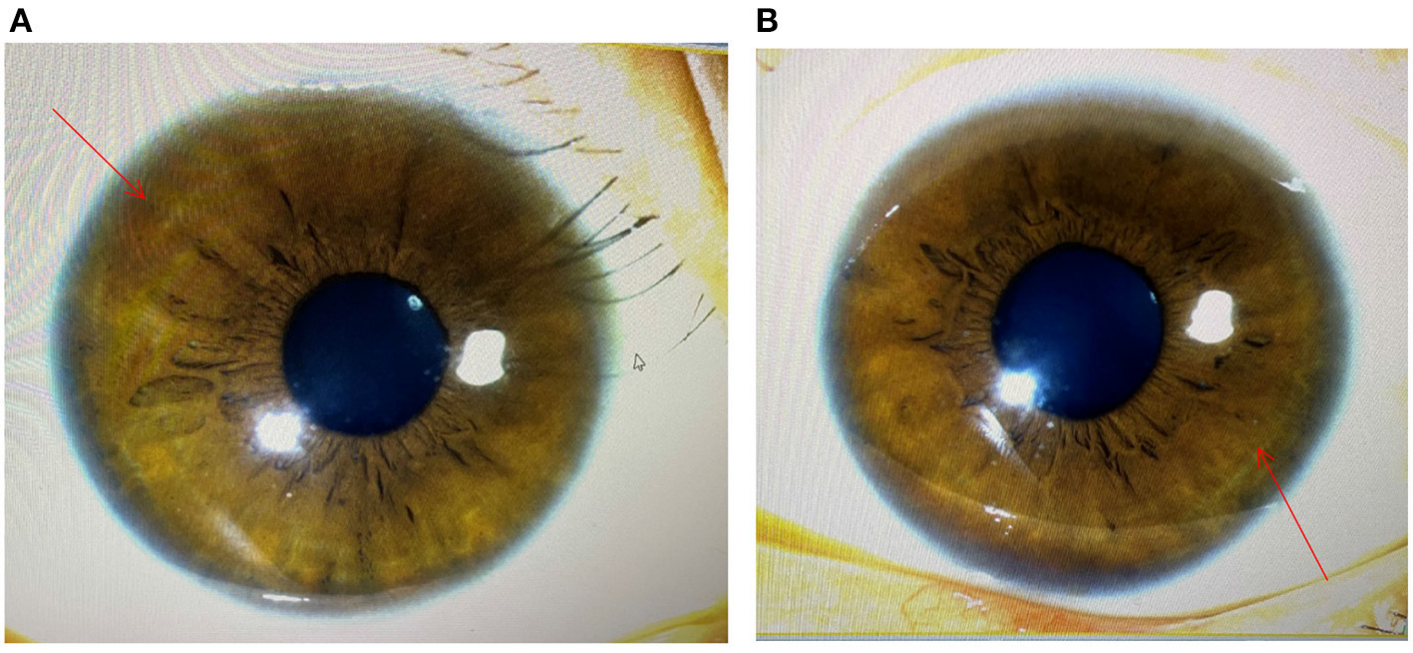
Image obtained during slit lamp examination showing characteristic Kayser–Fleischer (K–F) ring (arrowhead) at the margin of the cornea and scler. **(A)** the image of the left eye; **(B)** the image of the right eye.

The diagnosis of WD was established based on the presence of Coombs negative hemolytic anemia, Kayser-Fleischer rings on slit lamp examination (Figure 1), low level of serum ceruloplasmin, and obviously elevated levels of 24-h urine copper (3,804 μg/24 h) (Table 1). The whole exon region (about 20,000 genes) in the human genome sequencing was performed on the Illumina MiSeq next-generation sequencing (NGS) and Sanger sequencing. The former presented a possible loss of heterozygosity in the area of chr13q14.3 (52158814-53624916) with a length of 1.47 Mb. The latter revealed a novel heterozygote splicing mutation c.51+2T>G within intron 1 of the ATP7B (13q14.3|NM_000053.3) (Figure 1A), which was evaluated as likely pathogenic according to the American College of Medical Genetics and Genomics (ACMG) (10) criteria. The evidence of pathogenicity of the this mutation is PVS1+PM2+PP3+PP5.According to the scoring system developed at the 8th International Meeting on Wilson's disease, Leipzig 2001 (11), the patient received a score of 8 points in total, which further confirmed the diagnosis of WD.

Treatments included ornithine aspartate for preventing hepatic encephalopathy and albumin infusion combined with hydragogue for reducing ascites. Non-biological artificial liver treatment (plasmapheresis) was used to remove harmful substances and relieve hemolytic crisis and hepatic failure. For WD, the patient was treated with oral penicillamine (increased to 0.9 g/d) and zinc (0.15 mg/d). Plasmapheresis combined with chelation therapy was effective, because the ceruloplasmin level returned to the normal range and serum bilirubin and hemoglobin levels were normal on the day of her discharge. Notably, the patient's abdominal pain was relieved and the scattered papules on the scalp and forehead disappeared after a comprehensive treatment, which possibly suggested that photosensitivity and stomachache were symptoms of WD. However, the treatment of WD is life-long and should be continued after discharge to maintain normal liver functioning.

The patient had normal liver functioning during follow-up at 1 year after discharge, which was maintained by treatment with oral penicillamine (0.25 g). However, her liver function deteriorated after drug withdrawal without the authorization of her attending physicians at the latest follow-up; thus, she had to undergo liver transplantation. At the time of writing, specifically at 1.5 years after discharge, she is in a good condition (Table 1).

## Discussion

Prominent cirrhosis and hepatomegaly in this young patient on abdominal ultrasound suggested a long course of illness. Porphyria was considered first based on her outstanding symptoms of skin damage and stomachache. Porphyria, caused by the absence of enzyme activity in the heme biosynthetic pathway, leads to the increased concentration of porphyrin or its precursors (12). Some patients may present with chronic liver damage and cholelithiasis because of the excessive release of protoporphyrin into the biliary tract (13). Therefore, porphyrin accumulation in the liver and biliary tract leads to liver damage, ranging from minor liver biochemical abnormalities of elevated levels of bilirubin and transaminase to liver carcinoma (14). In addition, acute hepatic porphyrias present with episodic and acute neurovisceral symptoms (8), while photosensitivity may occur mainly in cutaneous porphyria (9). The clinical manifestations of porphyria hepatica are atypical and diversified. Specific diagnostic tests are few, and most of them show negative results. Therefore, the clinical misdiagnosis rate of WD is high. In this case, the diagnosis of porphyria was excluded using gene sequencing. However, attention must be given to such confounding manifestations to avoid misdiagnosis.

Studies focusing on characterizing mutations within the *ATP7B* gene have soared recently. Approximately 1019 different mutations have been reported in the *ATP7B* gene from the Human Genome Organization database (http://www.hgmd.cf.ac.uk/ac/index.php), updated in April, 2019. Among them, single-nucleotide missense/nonsense mutations are the most common, followed by insertions/deletions, and a few of splice site mutations. WD gene mutations are with significant geographic variations. The p.His1069Gln is found mostly in Northern America (15) and Europe (16), while the p.Arg778Leu is the most common in East Asians (17, 18). WD presents multiple phenotypic manifestations due to the combined action of genotype, diet, and environment (19). Multiple previous studies on the association between genotypes and clinical features showed that p.Arg778Leu is associated with a young age of onset and low levels of ceruloplasmin and serum copper, and both p.Arg919Gly and p.Thr935Met indicate high levels of ceruloplasmin (20). Another research suggested that a p.H1069Q variant is related to late onset and neurologic presentation of WD (21).

In the present case, the pathogenic mutation c.51 + 2T > G and loss of heterozygosity are compound heterozygotes on the two chromosomes of the proband, inherited from her father and new mutation of her own. The mutation c.51 + 2T > G is a new mutation that was first identified in 2019. The mutation is located in intron one, which predicted results in exon skipping, leading to a disorder of the encoded protein and loss of its normal function (22). As a result, *ATP7B* ATPase cannot be encoded, and copper accumulates. Hence, it could be classified as “pathogenic variants” of WD according to ACMG Standards and Guidelines (10). It has been confirmed previously that splice variants can alter the order of intron removal, thereby leading to exon skipping (23). One study had found that a splice mutation (c.561-3T>C) of intron 6, in the *POC1B* gene, in which exon 6 is partly skipped (24). Based on the findings reported in the literature (25) and those of our case, mRNA sequencing analysis revealed that deep intron deletion on both sides of the affected exon can cause exon jumping, intron retention, or sequence insertion of other genes in mRNA, and intron deletion may be a pathogenic mutation.

In addition, this patient showed special symptoms of skin damage, photosensitivity, and acute neurovisceral manifestations, which may be a special phenotype of the gene mutation (c.51 + 2T > G). For nonspecific skin changes, some features of hyperpigmentation, xerosis, acanthosis nigricans, and dermatomyositis have been reported in patients with WD (26). In a study involving children aged 4–17 years, Muammer et al. found that xerosis was common (45.7%) in relatively newly diagnosed children with WD, followed by keratosis pilaris (10.8%) and spider angioma (10.8%) (27). In an early study, hyperpigmentation was known to be related to WD. Histologically, it was found that hyperpigmentation of the skin was due to excessive melanin deposition rather than the presence of copper or iron (28). It had been confirmed that the skin changes are caused by the treatment with penicillamine, as its cutaneous side-effects include degenerative dermatoses, including cutis laxa, anetoderma, elastosis perforans serpiginosa, and lymphangiectasis (29, 30). Obviously, the skin lesion of this patient was not caused by adverse drug reactions, because of the first diagnosis. Most of the patients with WD have copper metabolic abnormalities with liver dysfunction. It seems likely that the liver abnormality plays an important role in the development of skin pigmentation in patients with WD. Thus, far, the exact pathophysiological mechanism of varied skin lesions in WD is not clear. Additionally, for acute neurovisceral manifestation, one patient presented with mild pancreatitis, which was attributed to copper deposition in the pancreas (31). The pigmented gallstone pancreatitis and cholangitis with concomitant obstructive jaundice have also been reported as the features of WD (6). A study involving 10 WD patients with different causes included three cases showed that acute pancreatitis is a complication of massive hemolysis, with a prevalence of ~25% (32). The cause of WD presenting as pancreatitis, thus, was presumed to be copper deposition or hemolysis. Therefore, further investigation is still needed to verify the causality between genotype and phenotype, especially the novel mutation c.51 + 2T > G and skin manifestations and acute neurovisceral symptoms. Unfortunately, few studies have reported a splice site variant of c.51 + 2T > G.

**Figure 2:**
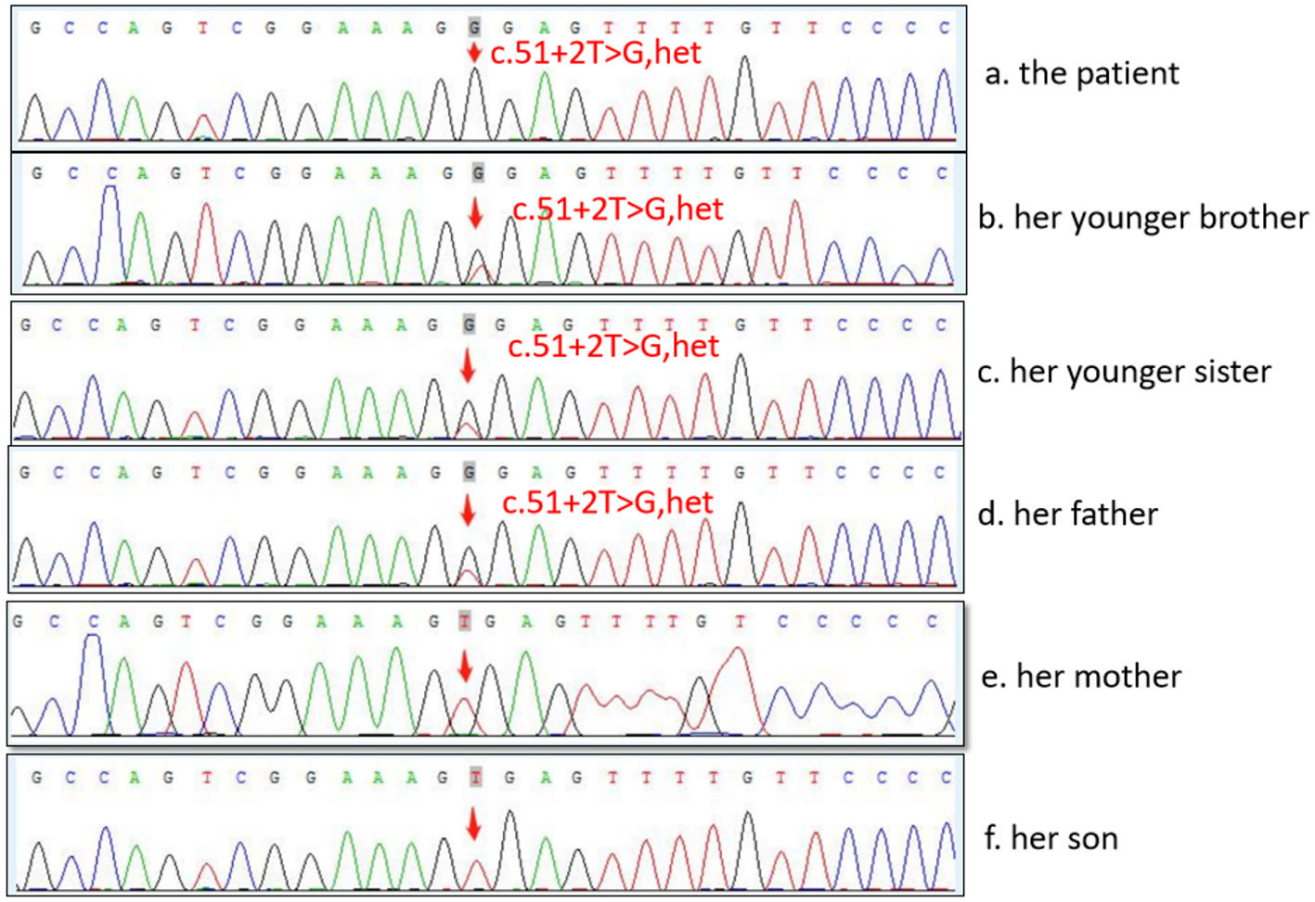
Sequence analysis of *ATP7B* gene in the family.

**Figure 3:**
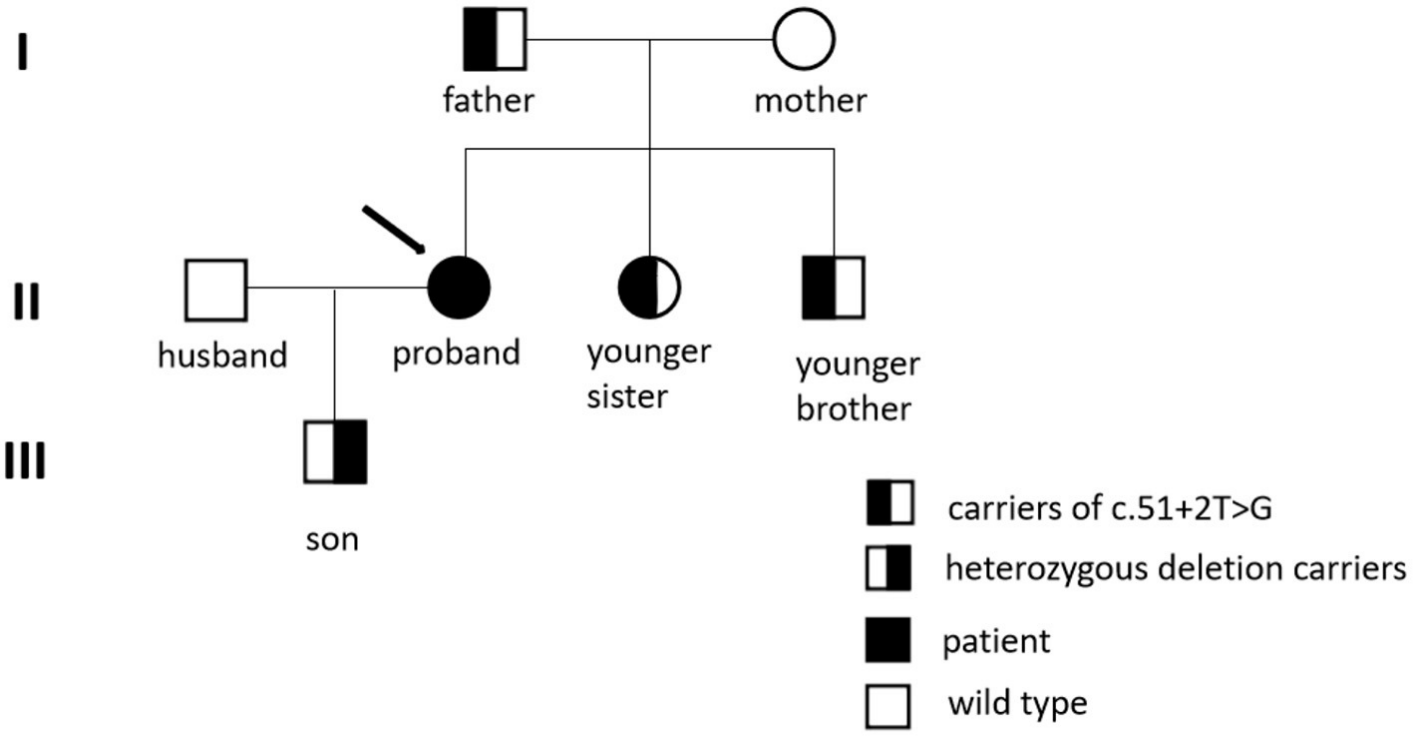
Family pedigree of the mutation in c.51+2T>G and heterozygous deletion.

First-degree relatives of the proband must be screened for WD; a higher (4.08%) than expected (0.5%) frequency of WD among their subsequent generations was detected (33). The parents, younger sister, and baby boy of the patient all underwent genetic testing, as shown in Figure 2. The c.51 + 2T > G variant located on intron one was detected in both the father and younger sister of the patient, and the same heterozygous deletion in the chr13q14.3 (52158814-53624916) region was located in her son. In this case, the pathogenic gene of the splicing mutation c.51 + 2T > G in intron one came from her father, while the new heterozygosity deletion originated from herself, which led to the formation of complex heterozygotes. Based on the phenotype of their families, we mapped the family pedigree, as shown in Figure 3. Although, none of her first-degree relatives had any pathognomonic physical sign of the disease, a study showed that prophylactic therapy was effective in WD, whereas a long period must elapse before the illness could be prevented in these asymptomatic families (34). We suggest that her family should regularly undergo liver function tests and should note any neuropsychiatric symptoms of WD. A low-copper diet and zinc therapy have been used successfully in asymptomatic or presymptomatic individuals (3).

The limitations in our report are quite obvious. It was not a controlled study, and only one patient with WD with splicing variants of c.51 + 2T > G in the *ATP7B* gene was involved. Thus, our findings may not be generalizable to other patients with WD. However, we have added a data comparison with a WD patient with other mutation (p.R778L) in Table 1. Although this mutation has been reported previous studies, more animal experiments with greater scientific rigor are needed to confirm the relationship between genotype and phenotype.

## Conclusion

In this case, the diagnosis of porphyria was excluded using gene sequencing. However, attention must be given to such confounding manifestations to avoid misdiagnosis. In this case report, we identified the rare splicing variants of c.51 + 2T > G in the *ATP7B* gene that may be involved in the pathogenesis of WD.The patient developed skin changes and stomachache, which have never been reported in WD populations. We hope that our case report may serve as a useful reference for generalists and specialists in hepatology or neurology in diagnosing the rare clinical characteristics of WD, to prevent misdiagnosis and for the early diagnosis and treatment of the condition.

## Data Availability Statement

The medical records and laboratory findings of this patient reported in the current study are available from the corresponding author on reasonable request.

## Ethics Statement

Written informed consent was obtained from the individuals for the publication of any potentially identifiable images or data included in this article.

## Author Contributions

JZ and Y-HW contributed to the design of this case report, recorded the medical information, and drafted the article. LW and R-CC analyzed the data and made revisions of the manuscript. All authors contributed to the critical revision and provided final approval of the submitted version of this article.

## Conflict of Interest

The authors declare that the research was conducted in the absence of any commercial or financial relationships that could be construed as a potential conflict of interest.

## Publisher's Note

All claims expressed in this article are solely those of the authors and do not necessarily represent those of their affiliated organizations, or those of the publisher, the editors and the reviewers. Any product that may be evaluated in this article, or claim that may be made by its manufacturer, is not guaranteed or endorsed by the publisher.

## References

[1] Terada K Schilsky LM Miura N Sugiyama T. Molecules in focus ATP7B (WND) protein. Int J Biochem Cell Bio. (1998) 30:1063–7. 10.1016/S1357-2725(98)00073-9 9785470

[2] Menkes JH. Menkes disease and Wilson disease: two sides of the same copper coin Part II: Wilson disease. Eur J Paediatr. (1999) 3:245–53. 10.1016/S1090-3798(99)90979-0 10595669

[3] Ala A Walker AP Ashkan K Dooley JS Schilsky ML. Wilson's disease. Lancet. (2007) 369:397–408. 10.1016/S0140-6736(07)60196-2 17276780

[4] Xie J-J Wu Z-Y. Wilson's Disease in China. Neurosci Bull. (2017) 33:323–30. 10.1007/s12264-017-0107-4 28265897 PMC5567514

[5] Kapoor N Cherian KE Sajith KG Thomas M Eapen CE Thomas N. Renal tubular function, bone health and body composition in Wilson's disease: a cross-sectional study from India. Calcif Tissue Int. (2019) 105:459–65. 10.1007/s00223-019-00588-z 31317233

[6] Nussinson E Shahbari A Shibli F Chervinsky E Trougouboff P Markel A. Diagnostic challenges of Wilson's disease presenting as acute pancreatitis, cholangitis, and jaundice. World J Hepatol. (2013) 5:649–53. 10.4254/wjh.v5.i11.649 24303094 PMC3847949

[7] Zhu Q Zhu K Wang J Bian W Lu J. Relationship between genetic mutations and clinical phenotypes in patients with Wilson disease. Medicine. (2019) 98:e18284. 10.1097/MD.0000000000018284 31804371 PMC6919422

[8] Wang B Rudnick S Cengia B Bonkovsky HL. Acute hepatic porphyrias: review and recent progress. Hepatol Commun. (2019) 3:193–206. 10.1002/hep4.1297 30766957 PMC6357830

[9] Christiansen AL Aagaard L Krag A Rasmussen LM Bygum A. Cutaneous porphyrias: causes, symptoms, treatments and the danish incidence 1989-2013. Acta Derm Venereol. (2016) 96:868–72. 10.2340/00015555-2444 27139922

[10] Richards S Aziz N Bale S Bick D Das S Gastier-Foster J. Standards and guidelines for the interpretation of sequence variants: a joint consensus recommendation of the American college of medical genetics and genomics and the association for molecular pathology. Genet Med. (2015) 17:405–24. 10.1038/gim.2015.30 25741868 PMC4544753

[11] Heathcote EJ Marcellin P Buti M Gane E De Man RA Krastev Z. European association for study of the liver. EASL clinical practice guidelines: Wilson's disease. J Hepatol. (2012) 56:671–85. 10.1016/j.jhep.2011.11.007 22340672

[12] Puy H Gouya L Deybach J-C. Porphyrias. Lancet. (2010) 375:924–37. 10.1016/S0140-6736(09)61925-5 20226990

[13] Baravelli CM Sandberg S Aarsand AK Tollanes MC. Porphyria cutanea tarda increases risk of hepatocellular carcinoma and premature death: a nationwide cohort study. Orphanet J Rare Dis. (2019) 14:77. 10.1186/s13023-019-1051-3 30944007 PMC6448269

[14] Singal AK. Porphyria cutanea tarda: recent update. Mol Genet Metab. (2019) 128:271–81. 10.1016/j.ymgme.2019.01.004 30683557

[15] Shah AB Chernov I Zhang HT Ross BM Das K Lutsenko S. Identification and analysis of mutations in the Wilson disease gene (ATP7B): population frequencies, genotype-phenotype correlation, and functional analyses. Am J Hum Genet. (1997) 61:317–28. 10.1086/514864 9311736 PMC1715895

[16] Ferenci P. Regional distribution of mutations of the ATP7B gene in patients with Wilson disease: impact on genetic testing. Hum Genet. (2006) 120:151–9. 10.1007/s00439-006-0202-5 16791614

[17] Dong Y Ni W Chen W-J Wan B Zhao G-X Shi Z-Q. Spectrum and classification of ATP7B variants in a large cohort of chinese patients with Wilson's disease guides genetic diagnosis. Theranostics. (2016) 6:638–49. 10.7150/thno.14596 27022412 PMC4805659

[18] Tatsumi Y Hattori A Hayashi H Ikoma J Kaito M Imoto M. Current state of Wilson disease patients in central Japan. Intern Med. (2010) 49:809–15. 10.2169/internalmedicine.49.2931 20453399

[19] Czlonkowska A Gromadzka G Chabik G. Monozygotic female twins discordant for phenotype of Wilson's disease. Mov Disord. (2009) 24:1066–88. 10.1002/mds.22474 19306278

[20] Cheng N Wang H Wu W Yang R Liu L Han Y. Spectrum of ATP7B mutations and genotype-phenotype correlation in large-scale chinese patients with Wilson disease. Clin Genet. (2017) 92:69–79. 10.1111/cge.12951 27982432

[21] Stapelbroek JM Bollen CW van Amstel JK van Erpecum KJ van Hattum J van den Berg LH. The H1069Q mutation in ATP7B is associated with late and neurologic presentation in Wilson disease: results of a meta-analysis. J Hepatol. (2004) 41:758–63. 10.1016/j.jhep.2004.07.017 15519648

[22] Chen Y-C Yu H Wang R-M Xie J-J Ni W Zhang Y. Contribution of intragenic deletions to mutation spectrum in chinese patients with Wilson's disease and possible mechanism underlying ATP7B gross deletions. Parkinsonism Relat Disord. (2019) 62:128–33. 10.1016/j.parkreldis.2019.01.001 30655162

[23] Takahara K Schwarze U Imamura Y Hoffman GG Toriello H Smith LT. Order of intron removal influences multiple splice outcomes, including a two-exon skip, in a COL5A1 acceptor-site mutation that results in abnormal pro-alpha1(V) N-propeptides and Ehlers-Danlos syndrome type I. Am J Hum Genet. (2002) 71:451–65. 10.1086/342099 12145749 PMC379186

[24] Weisschuh N Mazzola P Bertrand M Haack TB Wissinger B Kohl S. Clinical characteristics of POC1B-associated retinopathy and assignment of pathogenicity to novel deep intronic and non-canonical splice site variants. Int J Mol Sci. (2021) 22:5396. 10.3390/ijms22105396 34065499 PMC8160832

[25] Baskin B Gibson WT Ray PN. Duchenne muscular dystrophy caused by a complex rearrangement between intron 43 of the DMD gene and chromosome 4. Neuromuscul Disord. (2011) 21:178–82. 10.1016/j.nmd.2010.11.008 21134752

[26] Czlonkowska A Litwin T Dusek P Ferenci P Lutsenko S Medici V. Wilson disease. Nat Rev Dis Primers. (2018) 4:21. 10.1038/s41572-018-0018-3 30190489 PMC6416051

[27] Seyhan M Erdem T Selimoglu MA Ertekin V. Dermatological signs in Wilson's disease. Pediatr Int. (2009) 51:395–8. 10.1111/j.1442-200X.2008.02766.x 19400828

[28] Leu ML Strickland GT Wang CC Chen TSN. Skin pigmentation in Wilson's disease. JAMA. (1970) 211:1542–3. 10.1001/jama.1970.03170090058016 5467055

[29] Na SY Choi M Kim MJ Lee JH Cho S. Penicillamine-induced elastosis perforans serpiginosa and cutis laxa in Wilson's disease. Bri J Dermatol. (2000) 142:560–91. 10.1046/j.1365-2133.2000.03379.x 21165224 PMC2991731

[30] Goldstein JB McNutt NS Hambrick GW Hsu A. Penicillamine dermatopathy with lymphangiectases. Arch Dermatol. (1989) 125:92–7. 10.1001/archderm.1989.01670130094014 2642686

[31] Weizman Z Picard E Barki Y Moses S. Wilson's disease associated with pancreatitis. J Pediatr Gastroenterol Nutr. (1988) 7:931–3. 10.1097/00005176-198811000-00024 3199280

[32] Druml W Laggner AN Lenz K Grimm G Schneewei B. Pancreatitis in acute hemolysis. Ann Hematol. (1991) 63:39–41. 10.1007/BF01714959 1715192

[33] Dziezyc K Litwin T Chabik G Gramza K Czlonkowska A. Families with Wilson's disease in subsequent generations: clinical and genetic analysis. Mov Disord. (2014) 29:1828–32. 10.1002/mds.26057 25327413

[34] Sternlieb I Scheinberg IH. Prevention of Wilson's disease in asymptomatic patients. New Eng J Med. (1968) 278:352–9. 10.1056/NEJM196802152780702 5635646

